# 2786. Characterizing Ertapenem Neurotoxicity: A Systematic Review

**DOI:** 10.1093/ofid/ofad500.2397

**Published:** 2023-11-27

**Authors:** Hayato Mitaka, Rupali Jain, Robert M Rakita, Paul S Pottinger

**Affiliations:** University of Washington, Seattle, Washington; University of Washington Medical Center-Montlake, Seattle, WA; University of Washington, Seattle, Washington; University of Washington School of Medicine, Seattle, WA

## Abstract

**Background:**

Ertapenem is frequently used to treat ESBL-producing Enterobacterales infections. Although ertapenem has the highest incidence of delirium reported among antibiotics evaluated in a pharmacovigilance study of the FDA adverse event reporting system, little is known about clinical characteristics of its neurotoxicity. In this systematic review, we synthesize the existing literature on ertapenem neurotoxicity, focusing on comorbidities, clinical manifestations, and outcomes.

**Methods:**

We searched PubMed and EMBASE for all peer-reviewed articles from inception through April 2023 using the following search terms: "ertapenem" AND ("Neurotoxicity" OR "Encephalopathy" OR "Seizures" OR “status epilepticus” OR "Convulsions" OR "Delirium" OR "Psychosis" OR "Hallucinations" OR “altered mental status” OR coma OR stupor OR obtundation). Studies were included if they described ertapenem specifically and reported clinical data related to neurologic adverse events.

**Results:**

A total of 651 articles were identified through the initial database search and subsequent manual search. After removing duplicated items and screening based on title and abstract, 43 articles were assessed for eligibility. Finally, 30 studies with 114 patients were included in our data analysis. Of the 114 cases found, the mean age was 73, and 67% were male. The most common clinical signs and symptoms were seizures (69%), altered mental status/delirium/cognitive impairment (27%), and hallucinations (18%). The most common electroencephalogram (EEG) finding was slow waves, observed in 59%. Median time to onset was 4 days (IQR, 3–7 days) after starting ertapenem. Complete resolution of the symptoms was observed in 84% after discontinuation of ertapenem. Median time to recovery was 6.5 days (IQR, 3–8 days). Urinary tract infection was the most common indication for ertapenem use (49%). Renal dysfunction and predisposing central nervous system condition were noted in 70% and 32% of the patients, respectively.

**Conclusion:**

This systematic review summarizes the current evidence and clinical characteristics of ertapenem neurotoxicity. Our study underscores the need for raising awareness among clinicians and a large observational study to accurately assess the true incidence, risk factors, and outcomes.

**Disclosures:**

**All Authors**: No reported disclosures

